# Circulating Levels of Sirtuin 4, a Potential Marker of Oxidative Metabolism, Related to Coronary Artery Disease in Obese Patients Suffering from NAFLD, with Normal or Slightly Increased Liver Enzymes

**DOI:** 10.1155/2014/920676

**Published:** 2014-06-17

**Authors:** Giovanni Tarantino, Carmine Finelli, Franco Scopacasa, Fabrizio Pasanisi, Franco Contaldo, Domenico Capone, Silvia Savastano

**Affiliations:** ^1^Department of Clinical Medicine and Surgery, Federico II University Medical School of Naples, Via Sergio Pansini 5, 80131 Naples, Italy; ^2^Centro Ricerche Oncologiche di Mercogliano, Istituto Nazionale Per Lo Studio E La Cura Dei Tumori “Fondazione Giovanni Pascale”, IRCCS, 83013 Mercogliano, Italy; ^3^Center of Obesity and Eating Disorders, Stella Maris Mediterraneum Foundation, C/da Santa Lucia, Chiaromonte, 80035 Potenza, Italy; ^4^Department of Biochemistry and Medical Biotechnology, Federico II University Medical School of Naples, Via Sergio Pansini 5, 80131 Naples, Italy; ^5^Department of Neuroscience, Unit of Clinical Pharmacology, Federico II University Medical School of Naples, Via Sergio Pansini 5, 80131 Naples, Italy

## Abstract

The present study shows low circulating levels of SIRT4 in obese patients with nonalcoholic fatty liver disease mirroring its reduced mitochondrial expression in an attempt to increase the fat oxidative capacity and then the mitochondrial function in liver and in muscle. SIRT4 modulates the metabolism of free fatty acids reducing their high circulating levels but, unfortunately, increasing ROS production. Great concentration of free fatty acids, released by adipose tissue, coupled with oxidative stress, directly results in endothelial dysfunction, early atherosclerosis, and coronary artery disease risk factor.

## 1. Introduction

Both obesity and the metabolic syndrome (MS) in developing countries lead to increased mortality closely associated with coronary artery disease (CAD). The molecular mechanism of obesity involves downregulation of genes not only in free fatty acid (FFA) oxidation [1] but also in muscle [2]. Sirtuin (SIRT) 4 is an ADP-ribosyltransferase of 59 kDa variably expressed in liver mitochondria and in skeletal muscle and is associated with homeostasis of glucose/lipid metabolism. In particular, experimental studies using SIRT4 expression in primary hepatocytes and myotubes have evidenced its role as negative regulator of the mitochondrial oxidative metabolism. Accordingly, SIRT4 knockdown leads to increased FFA oxidation in liver and in muscle [2]. On the other hand, SIRT4 decreases the amino acid-stimulated insulin secretion by inhibiting the glutamate dehydrogenase activity in pancreatic *β*-cells [3, 4]. Furthermore, SIRT4 expression is reduced in the rat model of insulin resistance [5] and low SIRT4 mRNA expression was found in peripheral blood leukocytes in human type 2 diabetes mellitus [6].

Unclassified nonalcoholic fatty liver disease (NAFLD) or hepatic steatosis (nonalcoholic HS), characterized by excess triglyceride (TG) storage in the liver, is a further expression of the MS [7] and is an independent predictor of CAD risk [8]. Obese people have increased intramuscular TG (IMTG) accumulation [9]. This fat deposition, due to altered production of mitochondrial ATP, is linked to IR [10]. Intramuscular fat increases the muscle echo intensity and can easily be evaluated by ultrasound [11]. Common carotid intima-media thickness (cIMT) is a functional and structural marker of the atherosclerotic process [12]. The results of a recent study highlight the negative correlation of myocardial triacylglycerol accumulation with calorimetric changes, that is, resting metabolic rate (RMR), as a novel biomarker of CAD risk, in the sense that reduced energy expenditure and oxygen consumption may provide novel risk factors of obesity-induced reduced energy generation for myocardial contractile function [13].

We aimed at investigating in a sample of obese individuals whether circulating levels of SIRT4 were correlated with anthropometric measures, metabolic and calorimetric profile, HS, low-grade chronic inflammation markers, and strict CAD risk factors, such as IMT.

## 2. Methods 

### 2.1. Population

This research was performed by screening 234 consecutive subjects referred to the out-patient Metabolic Unit, from June 2010 to September 2012, for established obesity (at least three years), who were on a balanced low calorie, low fat (25% of calories) diet (lasting at least three months) and were characterized by sedentary life style. This cross-sectional study was carried out according to the principles of the Declaration of Helsinki and informed written consent was obtained from each patient.

### 2.2. Enrollment Criteria

Of the initial 234 participants, 18 were excluded due to marked intestinal meteorism which made it difficult to perform abdominal ultrasound (first step to screening HS), 17 patients due to steroid therapy (eight for bronchial asthma, five for rheumatoid arthritis, two for neuritis, and two for inflammatory bowel disease), and 32 due to taking one or more drugs (i.e., aspirin, metformin, statins, and fibrates) known to possibly alter laboratory data. Sixteen others were excluded because they presented hepatic comorbidities in their medical history (HCV/HBV infection, alcohol abuse, and PBC); finally eleven subjects were considered drop-outs because they refused to undergo full laboratory-instrumental examinations. Of the 140 patients who formed the final study population, characterized by obesity and presence of HS, with normal or slight increase of liver enzymes, and for the most part also by IMTG, ten morbidly obese patients underwent bariatric surgery during which a liver biopsy was obtained. Twenty lean, apparently healthy, subjects (screened for the entire laboratory panel and instrumental parameters used for the obese individuals) were chosen as controls to set reference value of circulating SIRT4. Further criteria for diagnosing HS were any viral, autoimmune, and metabolic liver disease (Wilson disease, hemochromatosis, or antitrypsin deficiency), ruled out by appropriate testing, following the generally accepted diagnostic guidelines. Celiac disease was excluded by estimates of IgA antitissue transglutaminase antibodies. Alcohol abuse was ruled out, according to the DSM-IV diagnostic criteria, by means of screening tests such as Michigan Alcohol Screening Test (MAST) and Cut down, Annoyed, Guilty, and Eye opener (CAGE), as well as random tests for blood alcohol concentration and the use of a surrogate marker, for example, mean corpuscular volume. Patients on antihypertensive drugs maintained a balanced therapeutic regimen throughout the study.

### 2.3. Anthropometrics and Metabolic Profile

The three degrees of obesity (light, moderate, and severe) were established on the basis of BMI cut-off points of 30–34.9 and 35–39.9 and >40 kg/m^2^, respectively. MS was not categorized but every criterion was analyzed. Visceral obesity was identified by measuring WC at the midpoint between the lower border of the rib cage and the iliac crest. Hip circumference was measured around the widest part of the buttocks, with the tape parallel to the floor, and the waist-to-hip (W/H) ratio was calculated. IR status was determined by the homeostatic metabolic assessment (HOMA), which was assessed by the formula: fasting insulin (*μ*U/mL) × fasting glucose (mg/dL)/405. Moreover, as the repeated HOMA measurements presented high within person variability in obese patients, HOMA values were averaged on the basis of at least five determinations to avoid misclassification.

### 2.4. Indirect Calorimetry

RMR was measured by indirect calorimetry using a canopy system (V max 29 N, Sensor Medics, Anaheim, USA) in a quiet environment and with patients in the supine position for 30 min before measurement. After a 15–20 min adaptation to the instrument, oxygen consumption and carbon dioxide production were determined for 45 min. Energy expenditure was derived from CO_2_ production and O_2_ consumption with the appropriate Weir formula neglecting protein oxidation [14]. BMR, expressed as kcal/24 h, was adjusted for changes in fat-free mass (FFM), which was evaluated by single-frequency bioimpedance analysis obtaining a RMR/FFM ratio, expressed as kcal/24 h∗kg of body [15].

### 2.5. Ultrasound Evaluation

Ultrasonographic measurements were performed using an Esaote system (Genoa, Italy). Specifically, transverse scanning was performed to measure the subcutaneous adipose tissue (SAT) and visceral adipose tissue (VAT) using an eleven and 3.5 MHz linear probe convex probe, respectively. The SAT was defined as the thickness between the skin-fat interface and the linea alba, avoiding compression. The VAT was defined as the distance between the anterior wall of the aorta and the internal face of the rectoabdominal muscle perpendicular to the aorta, measured one cm above the umbilicus. When the aortic walls were not visualized as they were obscured by bowel gas, the Doppler scan was used. Spleen longitudinal diameter (SLD) as index of low-grade chronic inflammation [16] was chosen to evaluate spleen volume and was carried out by posterolateral scanning. Maximum length (the optically greatest overall longitudinal dimension obtained from one of the two poles) and craniocaudal length (the optically maximal transversal dimension intercepting one of the two poles) were measured; the resulting values were then averaged, since the two measurements do not always coincide. The classification of “bright liver” or HS was based on the following scale of hyperechogenicity: grade 0 = absent, grade 1 = light, grade 2 = moderate, and grade 3 = severe, pointing out the difference between the densities of the liver and the right kidney [17]. Technically, echo intensity can be influenced by many factors, particularly by gain intensity. To avoid confounding factors that could modify echo intensity and thus bias comparisons, mean brightness levels of both liver and right kidney cortex were obtained on the same longitudinal sonographic plane.

**Figure 1:**
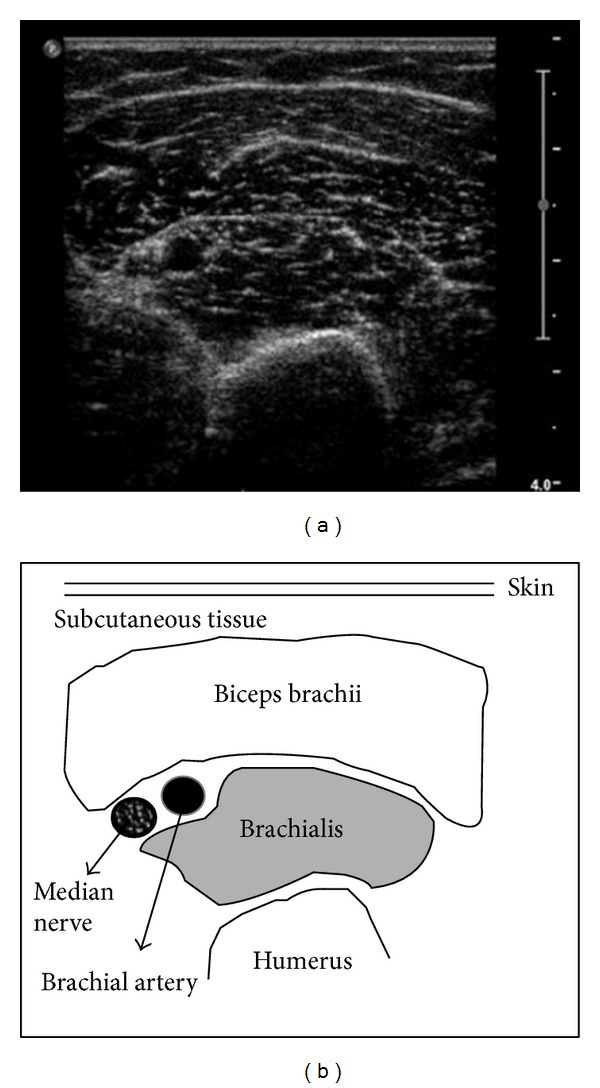
Skeletal muscle image. Echo intensity at ultrasound of a normal muscle (grade I) with related anatomic structures.

Muscle ultrasound, performed at the level of the biceps muscle of the left superior arm, is a convenient technique to visualize pathological muscle tissue, as it is noninvasive and provides results in real time. Both infiltration of fat and fibrous tissue increase muscle echo intensity; that is, the muscles become whiter on the ultrasound image [11]. To describe muscle echo intensity, Heckmatt and coworkers developed a visual grading scale in which grade I represented normal muscle (Figure 1) and grade IV represented a severely increased muscle echo intensity with total loss of bone echo (we chose biceps versus humerus, Figure 1) [18]. The levels of brightness of the liver and the biceps were calculated three times directly from the frozen images.

The common carotid, the carotid bulb, and the near and far wall segments of the internal carotid were scanned bilaterally. Subjects were examined in the supine position with the head turned 45° contralateral to the side of scanning. Images were obtained in longitudinal section, with a single lateral angle of isonation, optimizing the image for the far wall. cIMT was defined as the distance between the lumen-intima and the media-adventitia ultrasound interfaces. Measurements were performed off-line and consisted of six manual measurements at equal distances along 1 cm on the far wall of the common carotid. Left and right cIMT were averaged [19].

### 2.6. Blood Pressure Measurements

Systolic/diastolic arterial pressure (SBP, DBP) was the average of three consecutive readings taken by the physician during the day, during usual practice hours, after allowing the subjects to rest for five minutes in the sitting position.

### 2.7. Laboratory Data

Blood samples were drawn in fasting individuals. Serum triglycerides (TG), high density lipoprotein cholesterol (HDL), basal insulin, alanine aminotransferase (ALT), pseudocholinesterase (PCH), alkaline phosphatase (AP), gamma-glutamyl transpeptidase (gamma-GT), glycemia, and ferritin were performed by in-house standard procedures. C-reactive protein (CRP) values were determined by a high-sensitivity ELISA test, with reference values between 0.3 and 8.6 mg/L in healthy men and between 0.2 and 9.1 mg/L in healthy women (BioCheck, Inc., CA, USA). To have every laboratory test performed, venous blood samples from the antecubital vein were obtained following standard procedures. Blood was drawn into a 9 mL serum tube. Samples were centrifuged for 10 min at RCF of 850–1000 and freshly analyzed.

A part of sera was collected, aliquoted, and frozen at −20°C for the successive determination of SIRT4.

The kit for* in vitro* quantitative measurement of SIRT4 in human tissue homogenates and other biological fluids, based on a sandwich enzyme immunoassay, was provided by Uscn Life Science & Technology Company, 3603 Double Lake Dr., Missouri City, TX 77459. According to manufacturer's data, the detection range was 0.156–10 ng/mL. The standard curve concentrations used for the ELISA were 10 ng/mL, 5 ng/mL, 2.5 ng/mL, 1.25 ng/mL, 0.625 ng/mL, 0.312 ng/mL, and 0.156 ng/mL. To verify the reliability of the test, the generated standard curve was analyzed by linear regression. The *R* ^2^ was 0.9563. The sensitivity of this assay was defined as the lowest protein concentration that could be differentiated from zero. It was determined by adding two standard deviations to the mean optical density value of twenty zero standard replicates and calculating the corresponding concentration. The minimum detectable dose of human SIRT4 was less than 0.051 ng/mL. No significant cross-reactivity or interference between human SIRT4 and other sirtuins was observed. Intra-assay precision was determined as follows: three samples with low, middle, and high level human SIRT4 were tested 20 times on one plate. The interassay precision was weighed testing three samples with low, middle, and high level human SIRT4 on 3 different plates, 8 replicates in each plate. The coefficient of variation, calculated by SD/mean × 100 for the intra-assay and interassay, was <10% and <12%, respectively.

The minimum required total sample of population was calculated using the pooled standard deviation with the level test 0.05 and power 80% of SIRT4 in obese patients and controls.

### 2.8. Liver Histology

The diagnosis of NASH was made when three out of five criteria were proven by liver biopsy: steatosis, hepatocyte ballooning, lobular inflammation, portal inflammation, and Mallory bodies. Four criteria, measured by a 3-point scale, resulting in a sum score ranging from 0 to 12, were adopted. Specifically, the scores were as follows: steatosis: 0 = none; 1 = up to 33% of acini, mainly macrovesicular; 2 = 34–66% of acini, commonly mixed steatosis; 3 = over 66% of acini (panacinar), commonly mixed steatosis; hepatocyte ballooning: 0 = none; 1 = occasional in zone III; 2 = obvious in zone III; 3 = marked, predominantly in zone III; lobular inflammation: 0 = none; 1 = scattered neutrophils, occasional mononuclear cells, 1 or 2 foci per 20x objective; 2 = neutrophils associated with ballooned hepatocytes, mild chronic inflammation, 3 or 4 foci per 20x objective; 3 = acute and chronic inflammation, neutrophils possibly concentrated in zone III, over 4 foci per 20x objective. Portal inflammation was as follows: 0 = none; 1 = mild, some portal areas; 2 = mild to moderate, most portal areas; 3 = moderate to severe, most portal areas. Fibrosis was staged as follows: stage 0 = none; stage 1 = zone III perivenular, perisinusoidal (pericellular); stage 2 = stage 1 changes plus periportal fibrosis; stage 3 = bridging fibrosis; stage 4 = cirrhosis. NASH patients in the study required a sum score of at least 6 points.

### 2.9. Statistics

The NCCLS and Clinical and Laboratory Standards Institute guidelines C28-A2 and C28-A3 for estimating percentiles and their 90% confidence intervals (99%, double sided) were followed to calculate the reference limits of SIRT4 with the “robust method.”

Age, SIRT4, GH, TG, ferritin, *γ*-GT, ALT, CRP, HOMA, SBP, DBP, W/H ratio, and cIMT were not normally distributed when analyzed by Shapiro-Wilk (S-W) test, *P* < 0.05, and were expressed as median plus 25–75 interquartile range (IQR). Data for WC, SAT, VAT, RMR/FFM, HDL, BMI, fibrinogen, SLD, PCH, and AP, derived from a normally distributed population (S-W, *P* > 0.05), were articulated as mean plus SD. The Mann-Whitney (M-W) *U* test for independent samples was used when managing two populations to track differences of medians. The means of two populations were compared by *t*-test for independent sample. Frequencies were evaluated by chi-square. When comparing the grades of HS or IMTG, the ANOVA Kruskal-Wallis test, with the Conover-Inman test as post hoc analysis, was adopted. When the ANOVA analysis was adjusted for age, considering it as a covariate, the ANCOVA test was applied. Tracking the degree of association between variables, Spearman's rho for nonuniform intervals was used. To assess the independent effect of a quantitative variable on the prediction of another one, the linear regression analysis (least squares) was used, evaluating the coefficient with its standard error, 95% confidence intervals (CI), the *t* (*t*-stat), and *R* ^2^. A *t*-stat greater than 1.96 with significance less than 0.05 indicates that the independent variable is a significant predictor of the dependent variable within and beyond the sample. At multivariate analysis, the multiple regression (backward stepwise selection) was adopted, firstly entering all variables if *P* = 0.05 into the model and then removing, if *P* = 0.1, the nonsignificant variables sequentially, with a maximum number of 15 steps. The multicollinearity was set at the variance inflation factor and tolerance values of  >10 and <0.1, respectively. Similarly, to get the sense of which variables contribute more or less to the regression equation, the magnitude of standardized coefficient beta (*β*) was calculated. The factor analysis was applied to detect the structure in the relationships among variables, selecting a subset of variables having the highest correlations with the principal component factors. The Cattell Scree plot, with relative eigenvalues, was performed to screen the real factors. Extraction of the main components amounted to a variance maximizing (varimax) the rotation of the original variable space. To evaluate intra-/interobserver variability of the measurements, the mean difference in the measurements of the observers was first calculated. Next, the concordance correlation coefficient (*ρ*_c_), which measures precision and accuracy, was adopted to evaluate the degree of pair observations at ultrasound, with values >0.8 considered as indicators of good reliability. MedCalc, version 13 (MedCalc Software, Broekstraat 52, 9030 Mariakerke, Belgium) and SyStat 13 (Cranes Software International, Bangalore, India) were the packages used for the statistics.

## 3. Results

### 3.1. Prevalence

**Figure 2:**
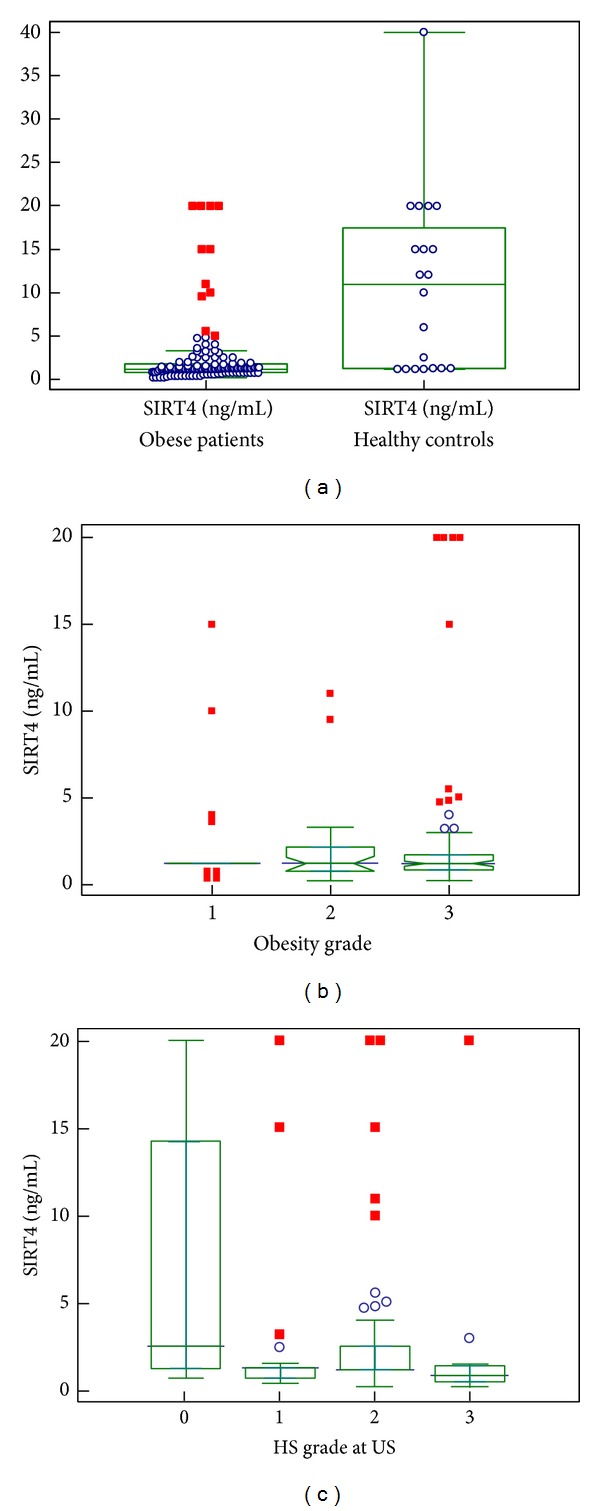
Behavior of SIRT4. (a) Behavior of circulating SIRT4 (ng/mL) in the whole population with the distribution of their serum levels; (b) circulating SIRT4 (ng/mL) according to grades of obesity; (c) circulating SIRT4 (ng/mL) according to severity of hepatic steatosis (HS).

Anthropometric, clinical, laboratory, ultrasound, and calorimetric data are summarized in Table 1. Serum levels of SIRT4 were measured in 140 obese patients and in 20 lean subjects without any metabolic alteration. The minimum required total sample was 106 subjects. The SIRT4 reference interval in healthy subjects was 0–31.8 ng/mL; circulating levels in obese patients were significantly lower than those present in healthy subjects, that is, 1.2 ng/mL (0.8–1.8) versus 11 ng/mL (1.2–15), median plus IQR, (M-W) *U* test, and *P* = 0.0001, without evident overlapping (Figure 2(a)). Six out of ten morbidly obese patients who underwent bariatric surgery patients were diagnosed as having NASH at liver histology.

Serum levels of SIRT4 were not significantly different between the three degrees of obesity (ANOVA Kruskal-Wallis, *P* = 0.9, Figure 2(b)). It should be stressed that the severely obese patients were largely represented in this study, if compared to those belonging to the groups with mild or moderate degree of obesity.

When obese subjects were subdivided into three groups on the basis of fat deposition in the liver (HS), detected at ultrasound, serum SIRT4 concentrations were significantly lower in obese subjects with severe grade of HS than in those with mild grade (ANOVA Kruskal-Wallis test, with post hoc analysis, *P* = 0.003, Figure 2(c)), not changing when the values were adjusted for age (ANCOVA, *P* = 0.007)

Obese subjects with severe IMTG, as revealed by ultrasound, did not show lower serum SIRT4 levels compared with milder grades of IMTG (ANOVA Kruskal-Wallis test, *P* = 0.5). Significant differences were found in serum SIRT4 concentrations between severely obese and mildly obese subjects when the values were adjusted for age (ANCOVA, *P* = 0.01)

### 3.2. Associations and Predictions

There was no correlation between serum SIRT4 levels and IR evaluated as HOMA (*P* = 0.1), TG concentrations (*P* = 0.4), inflammatory parameters, that is, SLD (*P* = 0.4), fibrinogen (*P* = 0.9), CRP (*P* = 0.2), ferritin (*P* = 0.1), hemodynamic indices, that is, SBP (*P* = 0.4), DBP (*P* = 0.7), and VAT at ultrasound of females (*P* = 0.5), and the calorimetric index, that is, RMR/FFM (*P* = 0.7). The grades of HS at ultrasound and the scores of steatosis at liver histology were well correlated (rho = 0.46, *P* = 0.035), and vice versa liver enzymes, that is, ALT (*P* = 0.9), PCH (*P* = 0.8), AP (*P* = 0.1), and *γ*-GT (*P* = 0.3), were not associated.

### 3.3. Univariate Analysis

**Figure 3:**
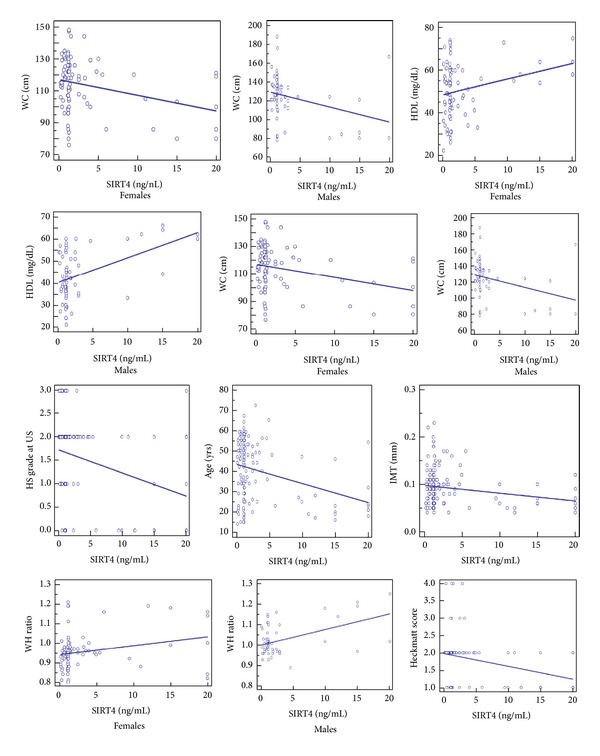
Predictions of SIRT4. Scatter diagram and regression lines. Waist circumference (WC); high density lipoprotein cholesterol (HDL); hepatic steatosis (HS) common carotid intima-media thickness (IMT); waist-to-hip (WH); ultrasound (US).

**Figure 4:**
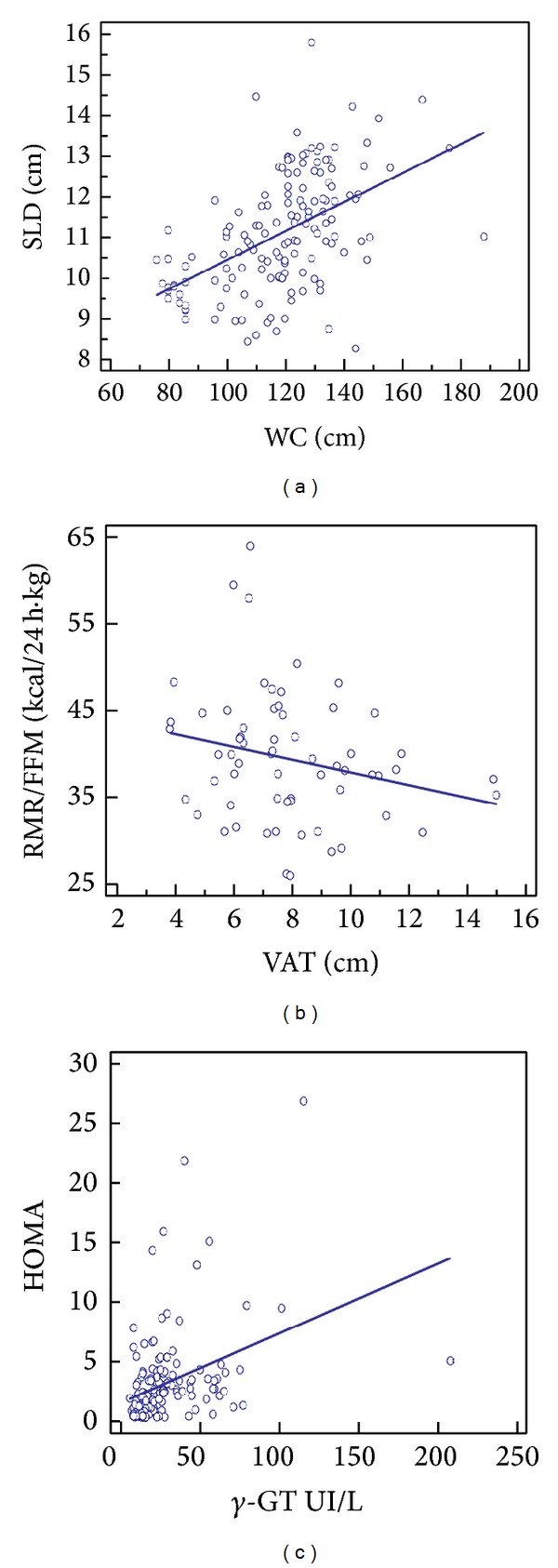
Other predictions. Scatter diagram and regression lines. Spleen longitudinal diameter (SLD); waist circumference (WC); resting metabolic rate/free fat mass (RMR/FFM); visceral adipose tissue (VAT); homeostatic metabolic assessment (HOMA).

The prediction of major severity IMT by lower SIRT4 levels was well documented and clearly evidenced in Table 2 and Figure 3. The prediction of SLD by WC was also demonstrated (*t* = 6.7, *R* ^2^ = 0.25, and *P* ≤ 0.0001, Figure 4). Equally, VAT was well-predicted RMR/FFM (*t* = 6, *R* ^2^ = 0.37, and *P* ≤ 0.0001, Figure 4), and *γ*-GT was substantially predicted by HOMA (*t* = 4.8, *R* ^2^ = 0.14, and *P* ≤ 0.0001, Figure 4). The prediction of HS grade by SLD and RMR/FFM was particularly evident (*t* = 6.4, *R* ^2^ = 0.22, and  *P* ≤ 0.0001 and *t* = −2.18, *R* ^2^ = 0.07, and  *P* = 0.03, resp.).

Other significant predictions of independent variables on circulating levels of SIRT4 were HDL of males and age, among the most significant ones, with an *R* ^2^ of 0.21 and 0.10, respectively. About age, also factor analysis confirmed its main role as risk factor for CAD (see below).

### 3.4. Multivariate Analysis

Among the classical cardiovascular risk factors such as age, hypertension, cIMT, IR, dyslipidemia, HS, and visceral obesity, at the multiple regression equation, only HDL (*β* = 0.285. *P* = 0.001) and age (*β* = −0.18, *P* = 0.038) were included in the model as independent predictors of SIRT4 levels, with IMT being not present due to the multicollinearity between it and the former ones.

### 3.5. Factor Analysis

According to Table 3, it revealed, as hidden relationships, that HOMA was strongly related to adiposity grade, that is, BMI, WC, VAT, and with markers of low-grade chronic inflammation, that is, CRP and SLD. Ferritin stood with WH ratio alone, not linked to other inflammatory markers. The latter parameters showed no correlation with the common carotid IMT increase. Finally, age, cIMT, SAP, and DAP shared the same behavior. The intra-/interobservational variability of the ultrasound estimations was not significant, with mean difference = 1.7, 2.2, 2.5, 2.3, and 1.9%, and 2.1, 3.3, 3.9, 4.6, and 3. 1% for the HS, VAT, SAT, SLD, and common carotid IMT, respectively, with a *ρ*_*c*_ of 0.92.

## 4. Discussion

The novel finding of this study was that lower serum levels of SIRT4 were present in obese subjects with HS and IMTG, independent of the severity of obesity. SIRT4 levels showed a strict relationship to some parameters reckoned as CAD risk factors, that is, low HDL, visceral obesity expressed as high W/H ratio but also as abundant VAT, evaluated by ultrasound, and finally increased cIMT. Of interest, the lowest SIRT4 levels were found in older obese males.

Trying to explain the finding that serum SIRT4 concentrations were significantly lower in obese subjects with severe grade of HS than in those with mild grade, we should take into account the following research data: (i) the increased degree of oxidative stress, due to decreased mitochondrial oxidative phosphorylation, initiating lipid deposition in the liver [20], (ii) the mitochondrial dysfunction, inducing apoptosis [21], mitochondrial permeability transition [22], and endoplasmic reticulum (ER) stress [23], (iii) the adjunct role of FFAs in inducing ER stress, leading to the progression of NAFLD [24], and (iv) the presence of reactive oxygen species (ROS) causing in turn the mitochondrial permeability transition pore to open [25]. ROS increase the inflammatory milieu that induces/worsens IR [26], a key mechanism of NAFLD. Since mitochondrial dysfunction in obese patients is not only the basis of NAFLD, but also of IMTG storage in skeletal muscles, increased blood concentrations of SIRT4 as a consequence of a sort of leakage of SIRT4 from mitochondria would be expected for detection. In contrast, our data did not confirm this expectation, although it is in line with the low SIRT4 expression in peripheral blood leukocytes of patients with type 2 diabetes mellitus [6].

We hypothesize that SIRT4 expression is likely diminished in the mitochondria in response to a high calorie diet and lack of exercise and is an attempt to decrease fat oxidative capacity reducing the mitochondrial ROS production in the liver and in muscle, but promoting the ectopic lipid storage, according to the hypothesis that mitochondrial functions are adjusted to meet environmental demands and maintain energy homeostasis [27].

The present study provided evidence that older male obese patients, characterized by the lowest serum SIRT4 combined with severe grade of HS and IMTG, were at greater risk than those with moderately low or normal SIRT4 levels. In other words, obese individuals with moderately low SIRT4 levels, due to disturbed muscle fat *β*-oxidation—a primary event in the etiology of obesity—were still able to provide a sufficient *β*-oxidation of FFAs that leads to less organ fat storage without forming excess ROS. In this context, the lack of associations between SIRT4 and HOMA as well as between markers of low-grade chronic inflammation and RMR/FFM—clue of reduced energy expenditure and oxygen consumption—lends credence to this hypothesis.

What was the link between SIRT4 and CAD risk? The obese phenotype, associated with great concentrations of FFAs released by adipose tissue, conveys endothelial dysfunction via oxidative stress [28], increased vascular endothelial factors, and nitric oxide imbalance. Visceral fat due to its close association with portal circulation has an additional role in endothelial dysfunction through hepatic IR. A collateral finding of this study was that visceral fat accumulation is associated with RMR [29] and is a significant predictor of HS, which in turn is related to CAD via low-grade chronic inflammation [19].

### 4.1. Limitations

A comparative tissue evaluation of SIRT4 expression or activity in hepatocyte or myocyte cultures was lacking. Equally, it would surely be of interest to have hepatic histology in every obese patient, but the lack of significant increase of liver enzymes avoided performing liver biopsy. Adiposity was not evaluated by the more precise MRI. Arterial stiffness could have represented a more valid choice to test the early atherosclerosis presence. Another limitation was the lack of clamp method that could have better clarified the association between serum SIRT4 levels and IR. Finally, it was evaluated neither the contextual variation of other sirtuins coordinating the oxidative capacity of tissue nor the presence of cross-reactivity with other sirtuins.

## 5. Conclusions

The finding of low circulating levels of SIRT4 deserves to be confirmed by other laboratory methods and by studies on larger populations, possibly including obese patients followed up for long periods.

## Figures and Tables

**Table 1:** Characteristics of the population.

Population (*n*)	Obese patients (140)	Healthy subjects (20)	Cross-sectional study
Parameter	Mean ± SD or median plus (25–75 IQR)	Mean ± SD or median plus (25–75 IQR)	Significance
Age (years)	46 (32–53)	23.5 (19–26)	*P* = 0.0001
Gender M/F	62	9	*P* = 0.9
Obesity degree I/II/III (*n*)	20/42/78	0	
BMI	42.1 ± 7.4	22.3 ± 1.1	*P* = 0.001
WC females cm	117.8 ± 13.2	82.7 ± 3.7	*P* = 0.001
WC males cm	131.6 ± 16	82 ± 2.8	*P* = 0.001
W-H ratio females	0.93 ± 0.05	1.17 ± 0.03	*P* = 0.001
W-H ratio males	1 ± 0.04	1.17 ± 0.04	*P* = 0.001
HS grade 1/2/3 US (*n*)	32/86/22	0	
IMTG score I/II/III/IV US (*n*)	34/96/4/6	0	
SIRT4 ng/mL	1.2 (0.8–1.8)	8 (1.2–15)	*P* = 0.0001
HOMA	2.62 (1.53–4.15)	0.43 (0.41–0.48)	*P* = 0.0001
Triglycerides mg/dL	128 (93.7–174.5)	103 (98–128)	*P* = 0.036
HDL females mg/dL	49.5 ± 12.9	59.5 ± 6.0	*P* = 0.025
HDL males mg/dL	40.0 ± 10.1	50 ± 4.3	*P* = 0.001
cIMT mm	0.1 (0.07–0.11)	0.06 (0.05-0.06)	*P* = 0.001
CRP mg/mL	0.52 (0.30–0.08)	0.12 (0.1–0.18)	*P* = 0.001
Fibrinogen g/L	310.7 ± 71.1	267.0 ± 33.7	*P* = 0.001
Ferritin females ng/mL	71.1 (21.5–72.29)	68.2 (26–81.3)	*P* = 0.4
Ferritin males ng/mL	116 (78–222.5)	108 (58.3–207.4)	*P* = 0.046
SLD cm	11.28 ± 1.4	9.8 ± 0.6	*P* = 0.001
RMR/FMM	39.5 ± 7.5	42 ± 3.8	*P* = 0.035
ALT U/L	27 (21–36.5)	16 (13–20)	*P* = 0.0001
Gamma-GT U/L	24 (16–36.2)	15.5 (11–17)	*P* = 0.0001
AP	75.9 ± 25.5	97.5 ± 6.7	*P* = 0.005
PCH	9375 ± 1793	8781 ± 1210	*P* = 0.17

Body mass index (BMI); waist circumference (WC); resting metabolic rate/free fat mass (RMR/FFM); high density lipoprotein cholesterol (HDL); common carotid intima-media thickness (IMT); waist-to-hip (WH); hepatic steatosis (HS) score; ultrasound (US); spleen longitudinal diameter (SLD), C-reactive protein (CRP); homeostatic metabolic assessment (HOMA); alanine aminotransferase (ALT); pseudocholinesterase (PCH); alkaline phosphatase (AP); gamma-glutamyl transpeptidase (gamma-GT).

**Table 2:** Independent variables predicting SIRT4 levels.

	Coefficient	Std. error	95% CI	*t*	*R* ^2^	*P*
WC females	−0.98	0.34	−1.6 to −0.30	**−2.9**	0.09	0.0050
WC males	−1.6	0.59	−2.7 to −0.42	**−2.7**	0.011	0.0086
HDL females	0.7	0.26	0.23 to 1.25	**2.9**	0.09	0.0053
HDL males	1.13	0.28	0.58 to 1.7	**4.1**	0.21	0.001
HS	−0.049	0.013	−0.077 to −0.02	**−3.5**	0.08	0.0005
Age	−0.94	0.23	−1.4 to −0.48	**4.0**	0.10	0.0001
cIMT	−0.0016	0.0006	−0.003 to −0.0004	**−2.7**	0.05	0.0075
W-H ratio	− 0.004	0.0011	0.006 to 0.002	**−3.2**	0.09	0.003
VAT at US males	−0.19	0.08	−0.36 to −0.03	**−2.4**	0.08	0.02
HeS	−0.026	0.01	−0.048 to −0.004	**−2.3**	0.04	0.02
BMI	−0.49	0.15	−0.80 to −0.19	**−3.2**	0.07	0.0016

Body mass index (BMI); waist circumference (WC); resting metabolic rate/free fat mass (RMR/FFM); visceral adipose tissue (VAT); high density lipoprotein cholesterol (HDL); common carotid intima-media thickness (cIMT); waist-to-hip (W-H); hepatic steatosis (HS) score; ultrasound (US); Heckmatt score (Hes).

**Table 3:** Rotated loading matrix (varimax, gamma = 1.0) of factor analysis.

Factor	1	2	3
HOMA	***0.827***	0.009	−0.275
SLD	***0.827***	0.009	−0.275
AGE	−0.075	0.263	***0.776***
RMM/FFM	−0.024	−0.076	−0.380
SIRT4	0.069	−0.584	−0.166
IMT	−0.059	0.292	***0.568***
VAT	***0.609***	0.012	0.180
SAT	0.384	−0.529	−0.056
DBP	0.202	−0.048	***0.533***
SBP	0.134	−0.285	***0.562***
Ferritin	0.110	***0.658***	−0.051
fibrinogen	0.349	−0.008	0.026
CRP	***0.519***	0.046	0.206
PCH	−0.115	0.318	0.100
Gamma-GT	0.287	0.301	0.210
ALT	0.313	0.248	−0.231
HDL	−0.220	−0.670	0.140
W-H ratio	0.078	***0.501***	0.168
WC	***0.773***	−0.017	0.202
BMI	***0.685***	−0.359	0.242

Percent of total variance explained by factors: 1 (17.8); 2 (11.8); 3 (10.6).

High density lipoprotein cholesterol (HDL); waist circumference (WC); common carotid intima-media thickness (cIMT); waist-to-hip (W-H); visceral adipose tissue (VAT); subcutaneous adipose tissue (SAT); systolic blood pressure (SBP); diastolic blood pressure (DBP); alanine aminotransferase (ALT); pseudocholinesterase (PCH); C-reactive protein (CRP); spleen longitudinal diameter (SLD); resting metabolic rate/free fat mass (RMR/FFM); homeostatic metabolic assessment (HOMA). The critical value was calculated by doubling Pearson's correlation coefficient for a 1% level of significance (5.152)/square root of the total population (140 + 20) minus 2, that is, 0.410.
