# Corrigendum to “Circulating Levels of Sirtuin 4, a Potential Marker of Oxidative Metabolism, Related to Coronary Artery Disease in Obese Patients Suffering from NAFLD, with Normal or Slightly Increased Liver Enzymes”

**DOI:** 10.1155/2018/6357164

**Published:** 2018-08-13

**Authors:** Giovanni Tarantino, Carmine Finelli, Franco Scopacasa, Fabrizio Pasanisi, Franco Contaldo, Domenico Capone, Silvia Savastano

**Affiliations:** ^1^Department of Clinical Medicine and Surgery, Federico II University Medical School of Naples, Via Sergio Pansini 5, 80131 Naples, Italy; ^2^Centro Ricerche Oncologiche di Mercogliano, Istituto Nazionale Per Lo Studio E La Cura Dei Tumori “Fondazione Giovanni Pascale”, IRCCS, 83013 Mercogliano, Italy; ^3^Independent Researcher, Naples, Italy; ^4^Department of Biochemistry and Medical Biotechnology, Federico II University Medical School of Naples, Via Sergio Pansini 5, 80131 Naples, Italy; ^5^Department of Neuroscience, Unit of Clinical Pharmacology, Federico II University Medical School of Naples, Via Sergio Pansini 5, 80131 Naples, Italy

In the article titled “Circulating Levels of Sirtuin 4, a Potential Marker of Oxidative Metabolism, Related to Coronary Artery Disease in Obese Patients Suffering from NAFLD, with Normal or Slightly Increased Liver Enzymes” [1], the second author, Carmine Finelli, is not affiliated to the Stella Maris Mediterraneum Foundation and should be listed as an independent researcher. The corrected list of affiliations is shown above.
